# Simultaneous multiple-excitation multiphoton microscopy yields increased imaging sensitivity and specificity

**DOI:** 10.1186/1472-6750-11-20

**Published:** 2011-03-02

**Authors:** Margaret T Butko, Mikhail Drobizhev, Nikolay S Makarov, Aleksander Rebane, Brendan C Brinkman, Joseph G Gleeson

**Affiliations:** 1 Departments of Neuroscience and Biomedical Graduate Program, Howard Hughes Medical Institute, University of California, San Diego, La Jolla, CA 92093 USA; 2 Department of Physics, Montana State University, Bozeman, MT 59717 USA

## Abstract

**Background:**

Multiphoton microscopy (MPM) offers many advantages over conventional wide-field and confocal laser scanning microscopy (CLSM) for imaging biological samples such as 3D resolution of excitation, reduced phototoxicity, and deeper tissue imaging. However, adapting MPM for critical multi-color measurements presents a challenge because of the largely overlapping two-photon absorption (TPA) peaks of common biological fluorophores. Currently, most multi-color MPM relies on the absorbance at one intermediate wavelength of multiple dyes, which introduces problems such as decreased and unequal excitation efficiency across the set of dyes.

**Results:**

Here we describe an MPM system incorporating two, independently controlled sources of two-photon excitation whose wavelengths are adjusted to maximally excite one dye while minimally exciting the other. We report increased signal-to-noise ratios and decreased false positive emission bleed-through using this novel multiple-excitation MPM (ME-MPM) compared to conventional single-excitation MPM (SE-MPM) in a variety of multi-color imaging applications.

**Conclusions:**

Similar to the tremendous gain in popularity of CLSM after the introduction of multi-color imaging, we anticipate that the ME-MPM system will further increase the popularity of MPM. In addition, ME-MPM provides an excellent tool to more rapidly design and optimize pairs of fluorescence probes for multi-color two-photon imaging, such as CFP/YFP or GFP/DsRed for CLSM.

## Background

Confocal laser scanning microscopy (CLSM) has become a standard imaging technique in molecular biology because it offers subcellular and three-dimensional spatial resolution, high temporal resolution, and the ability to resolve several fluorescent events simultaneously using multiple excitation lasers [1]. However, single-photon excitation used in CLSM results in broad exposure to high energy UV and visible excitation sources in the specimen above the focus plane, which can reduce the viability of photosensitive, living samples [2]. Multiphoton microscopy (MPM) has gained popularity because it offers decreased phototoxicity and increased sample viability while preserving the four-dimensional image resolution of CLSM due to its nonlinear excitation profile, which enables excitation predominantly at the focal point on the excitation plane [3-5]. MPM has also gained popularity for deep tissue imaging because the infrared (IR) light used to generate multiphoton excitation is scattered to a lesser extent in biological samples than higher energy visible or UV light [6,7].

However, CLSM still remains the most widely used technique for multi-color imaging because single-photon absorption peaks of common biological fluorophores are relatively narrow and usually non-overlapping. This characteristic makes it easy to differentially excite a set of spectrally distinct fluorophores in a given sample by simply incorporating standard excitation laser lines for multi-color CLSM. In contrast, two-photon absorption (TPA) spectra of common biological fluorophores are characterized by broad, overlapping absorption peaks that can result in off-peak excitation, thereby creating signal ambiguity [8,9]. Therefore, although MPM offers many advantages over CLSM, it has not replaced CLSM for multi-color measurements that are critical to most fields of biology.

The most popular multi-color MPM takes advantage of the broad, overlapping TPA peaks by selecting a single intermediate wavelength of excitation to excite multiple fluorophores simultaneously [4,10,11]. However, unlike CLSM, this multi-color MPM method often requires off-peak excitation of each fluorophore in order to excite all fluorophores in a given sample simultaneously, thereby decreasing the excitation efficiency and making excitation unequal over the given set of fluorophores [12]. In addition, finding the optimal intermediate wavelength for a given pair of fluorophores can be laborious and ultimately depends on the conditions of the experiment, [13] which makes it difficult to adopt a standard that can be used by other laboratories. Furthermore, adequate separation of the emission signal from multiple fluorophores often relies on the implementation of either very narrow variable band-pass filters (VBFs), which can significantly attenuate the true signal, or spectral scanning, which requires significantly longer exposure times [14,15]. These methods are not only expensive, but also reduce sample viability because of the increased excitation power or increased exposure times that are required to achieve sufficient, unambiguous signal in each emission channel.

We predicted that it should be possible to optimize an imaging system for dual color MPM image collection using multiple excitation wavelengths, similar to most standard CLSM systems. We tested common commercial antibody-linked fluorescent dyes for their two-photon excitation profiles and designed an imaging system to achieve high spectral separation of selected red and green fluorescent probes. We describe a novel multiple-excitation MPM (ME-MPM) system that is easily tunable for different pairs of fluorophores under different imaging conditions. Here we differentiate ME-MPM from single-excitation MPM (SE-MPM), the current standard in the field. ME-MPM demonstrated comparable temporal resolution, improved signal-to-noise ratios, and improved spectral separation of pairs of fluorophores without the use of VBFs or increased excitation power. In photosensitive living tissue, ME-MPM reduced the time required to set up optimal imaging parameters, leading to faster set up time and reduced exposure to light radiation before acquisition. We believe that this will allow for enhanced viability in photosensitive tissues as well as reduced photobleaching during imaging set up.

## Results and Discussion

### Multi-excitation MPM platform

**Figure 1:**
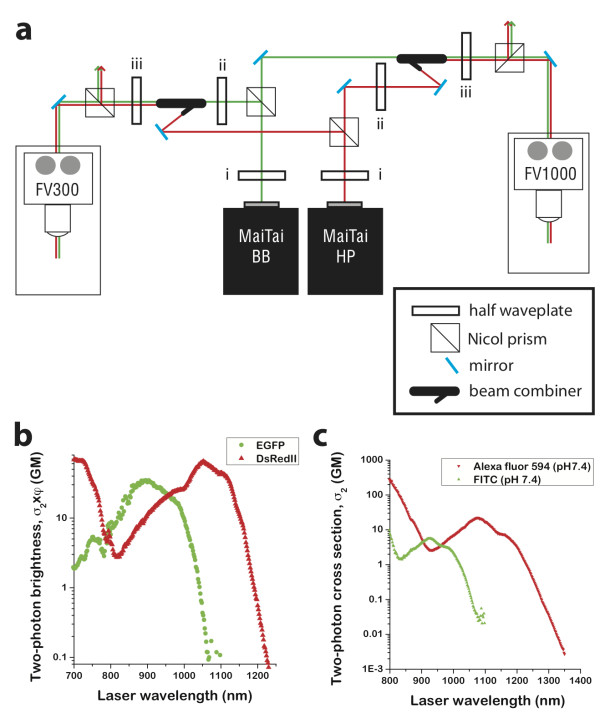
**The ME-MPM design**. (**a**) Optical setup for multiple-excitation multiphoton microscopy (ME-MPM) measurements. Beams from Mai Tai broad-band (BB) and Mai Tai high power (HP) sources were directed to either an upright confocal microscope (FV300) or an inverted confocal microscope (FV1000). Note that the Nicol prisms split the beam into two paths directed to either microscope for both lasers. The setting of each polarizer can be easily adjusted by rotating the preceding half-wave plate to direct more or less light toward each system, allowing for optimal excitation of each fluorophore during dual color imaging. The control of half-wave plates i, ii, and iii is described in the text and in Additional file 1. (**b**) Two-photon absorption (TPA) spectra for EGFP and DsRedII fluorescent proteins in Goeppert-Mayer units. (**c**) TPA spectra for the hydrazine derivatives of FITC (green), and Alexa Fluor 594 (red) in Goeppert-Mayer units. We do not present the spectra in terms of TPA brightness, *σ*_2 _× *φ*, where *φ *is the fluorescence quantum yield, because the quantum yields of these dyes are similar in the same environment and they are quite sensitive to pH and other conditions [22]. Imaging experiments presented here were performed at 920 nm for green-emitting fluorophores and at 1015 to 1040 nm for red-emitting fluorophores.

We designed an ME-MPM platform to direct two separate tunable excitation lasers into either of two confocal microscopes. ME-MPM was conducted on an upright Olympus confocal point scanning system equipped with FluoView 300 software (FV300) or an inverted Olympus spectral deconvolution confocal point scanning system equipped with variable band-pass filters (VBFs) and FluoView 1000 software (FV1000) using Spectra Physics Mai Tai broad-band (BB, 710 nm - 990 nm) and Mai Tai high-power (HP, 690 nm - 1040 nm) two-photon excitation sources (Figure 1a). This design allowed us to direct the light path to either an upright or an inverted stage without altering the optical setup between experiments.

The two excitation beams were independently modulated and were converged using a beam combiner and then directed into the microscope scan heads. The timing of the two excitation sources was random and two-pulse imaging may induce unexpected state-transitions, [16] which is not addressed here. In order to direct 100% of each excitation laser to only one microscope, we adjusted the polarization of the laser light coming from each excitation source using the first set of half-wave plates (i), such that only one beam exited the following Nicol prism. Once set, the first set of half-wave plates (i) remained fixed over the entire image acquisition period. We controlled the intensity of each excitation laser independently by adjusting the polarization of each excitation beam using a second half-wave plate (ii) before it entered the beam combiner, which attenuates the laser based on its polarization. This second set of half-wave plates (ii) was used to optimize the excitation of green or red fluorophores in the sample independently. Since the two excitation lasers exited the beam combiner orthogonal to each other, we controlled the ratio of green-to-red excitation light intensity using a third set of half-wave plates (iii) before directing the excitation light through a second fixed Nicol prism. This third set of half-wave plates (iii) was used to optimize the signal-to-noise in the green and red emission channels. A more detailed description of the optical path is described in Additional file 1. The power of excitation light achieved at the sample was measured from 690 nm to 1020 nm (Additional file 2). This design allowed us to carry out both ME-MPM and SE-MPM on either the upright or the inverted stage without modifying the optical table.

Two-photon absorption (TPA) spectra were collected for green and red dyes as well as for fluorescent proteins that are commonly used for imaging epitopes in fixed cells (Figure 1b-c) to identify non-overlapping regions of maximum absorption for each probe. Both FITC and EGFP exhibited local maxima in absorption between 900 nm and 920 nm, at which point Alexa Fluor 594 and DsRedII exhibited lower absorption. Therefore, light at 920 nm was used in ME-MPM experiments to excite green-fluorescing dyes and proteins. DsRedII exhibited a local maximum at 1040 and Alexa Fluor 594 exhibited a local maximum at 1080 nm, at which point the green-fluorescing dyes exhibited vanishing absorption. However, the IR excitation lasers in this setup were only tunable only up to 1040 nm. Therefore, 1040 nm was used to excite red-fluorescing dyes and proteins using the ME-MPM system, at which point green-fluorescing dyes exhibited very weak absorption. SE-MPM was performed at an empirically determined intermediate wavelength based on the fluorophores in the sample.

### Spectral separation in whole cells

**Figure 2:**
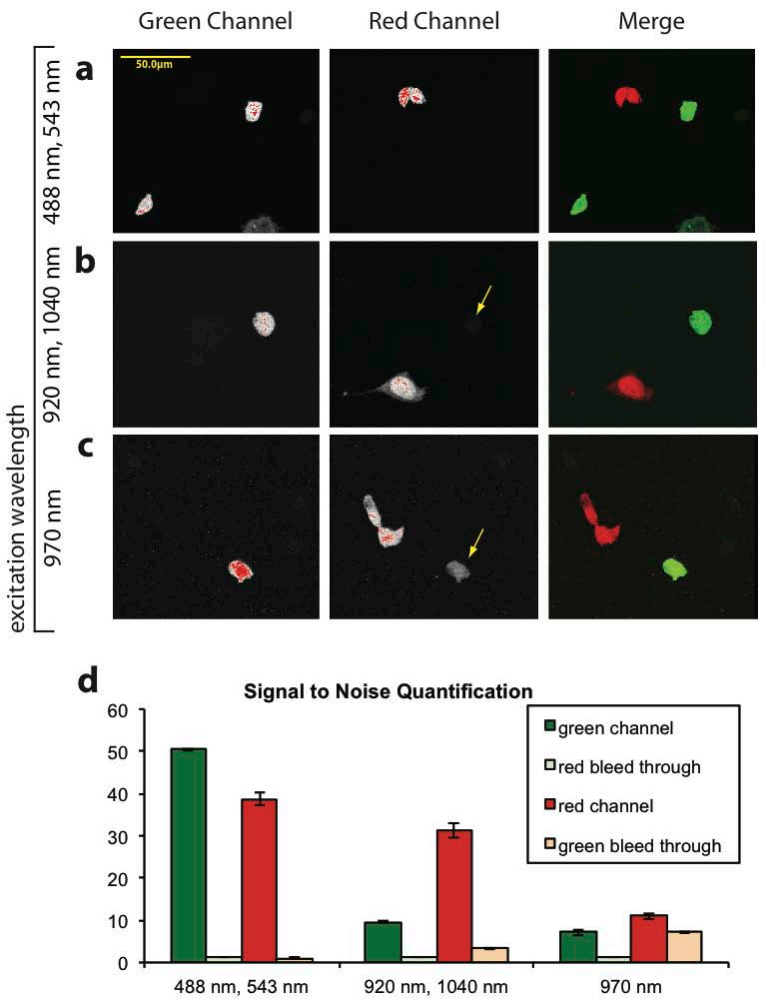
**ME-MPM improved signal-to-noise ratios in whole cells**. Images of 293T cells transfected with either hrGFP or DsRedII were collected using sequential single photon excitation at 488 nm and 543 nm for CLSM (**a**), simultaneous ME-MPM at 920 nm and 1040 nm (**b**), or SE-MPM at 970 nm (**c**). Signal intensity was held constant at the saturation point in the red channel over all experiments (**a-c)**. Note that there is more bleed-though from hrGFP signal into the red emission channel for SE-MPM than for either ME-MPM or CLSM (yellow arrows in **b-c**). Scale bar: 50 μm. All MPM images were acquired under identical VBF settings. The red and green emission signal generated from each excitation source in ME-MPM are shown in Additional file 3 and indicate reduced cross-excitation of the fluorophores in the sample. (**d**) Quantification of signal-to-noise ratios that were achieved for green signal and red bleed-through in the green emission channel as well as red signal and green bleed-through in the red emission channel.

To test whether ME-MPM increased the spectral resolution of two fluorophores in a sample compared to SE-MPM, we imaged combined pools of 293T cells expressing either cytoplasmic humanized renilla GFP (hrGFP) or DsRedII. Three dimensional z-stacks were collected on the FV1000 inverted confocal microscope using CLSM at 488 nm and 543 nm, ME-MPM at 920 nm and 1040 nm, and SE-MPM at 970 nm, which we empirically found to be the optimal intermediate excitation wavelength and which was also verified spectroscopically (Figure 1b and [9]) for this pair of fluorophores (Figure 2). ME-MPM at 920 nm and 1040 nm achieved comparable spectral separation of the green and red emitting fluorophores as sequential CLSM at 488 nm and 543 nm (Figure 2a-b). Under identical acquisition settings, we blocked one of the lasers at a time to verify that only one fluorophore was excited by each excitation source in ME-MPM. This excitation separation was comparable with what was achieved using sequential CLSM (Additional file 3).

Under identical VBF settings, SE-MPM at 970 nm achieved similar signal intensity as ME-MPM but showed increased cross-excitation and false-positive bleed-through in the emission channels, thereby adding ambiguity to the final image (Figure 2c). When the VBF settings were further narrowed to better separate green and red signals in the emission channels, a notable amount of signal was lost in both the red and green emission channels, resulting in poor image quality (data not shown). Therefore, ME-MPM demonstrated better spectral separation and higher signal-to-noise ratios over SE-MPM for green- and red-fluorescent proteins.

### Spectral resolution of subcellular structures

**Figure 3:**
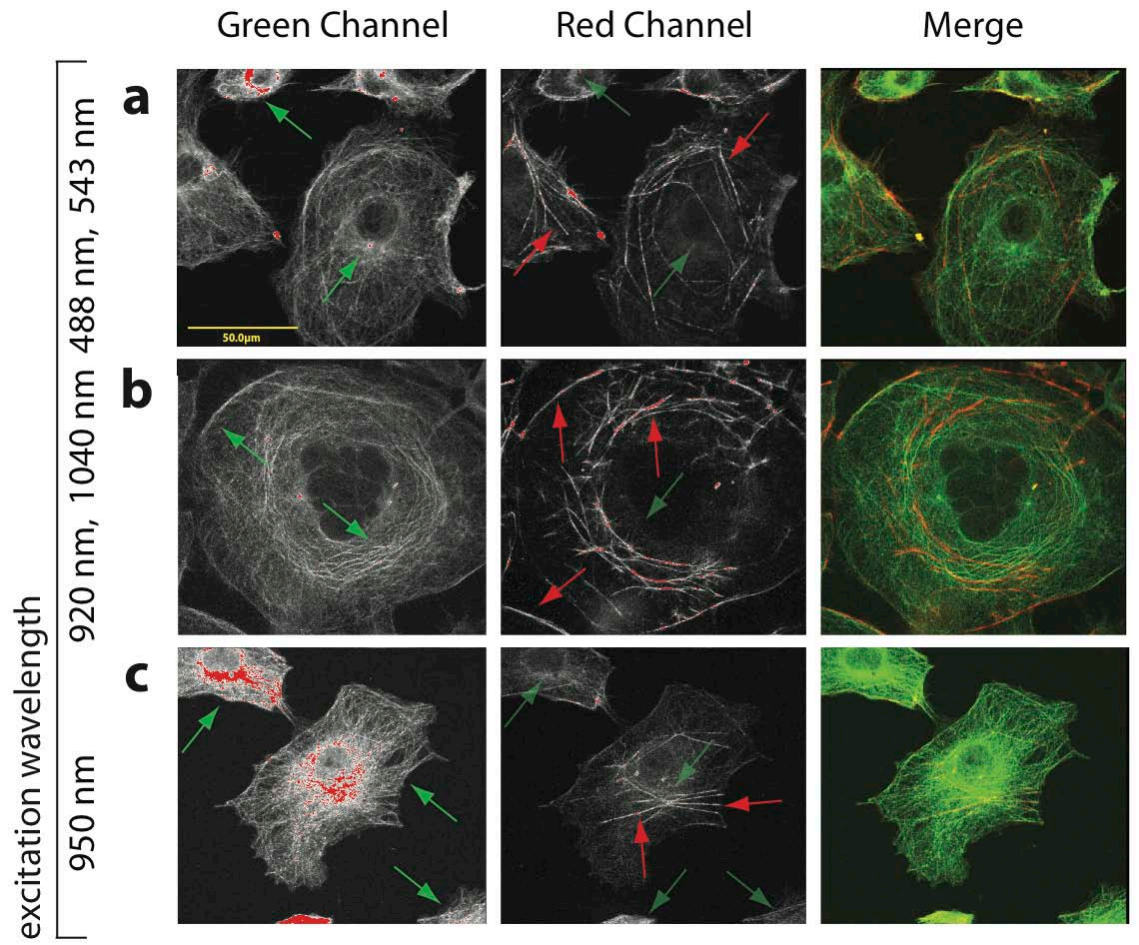
**ME-MPM improved signal-to-noise ratios and spectral separation in subcellular structures**. Images of fixed Cos7 cells stained with FITC-tubulin and Alexa Fluor 594-phalloidin were collected using simultaneous CLSM at 488 nm and 543 nm (**a**), simultaneous ME-MPM at 920 nm and 1040 nm (**b**), and SE-MPM at 950 nm (**c**). Green and red arrows correspond to distinct signal from FITC and Alexa Fluor 594, respectively, and dim green arrows correspond to bleed-through green signal into the red emission channel. Note that ME-MPM achieved comparable spectral separation to CLSM, whereas SE-MPM was hindered by false positive bleed-through of green emission signal in the red channel (middle panel, haze). Scale bar: 50 μm. The red and green emission signal generated from each excitation source in ME-MPM are shown in Additional file 3 and further verify that ME-MPM achieved better spectral separation by decreasing cross-excitation and false-positive bleed-through compared to SE-MPM.

To test whether ME-MPM could also be used to improve spectral separation in subcellular structures, we imaged Cos7 cells that were immunostained for tubulin (FITC) and actin (Alexa Fluor 594). Three dimensional z-stacks were collected on the FV300 upright microscope using simultaneous CLSM at 488 nm and 543 nm, ME-MPM at 920 nm and 1040 nm, and SE-MPM at 950 nm, which we empirically found to be the optimal intermediate excitation wavelength and which also roughly corresponds to the intersection point of the TPA spectra (Figure 1c) for this pair of fluorophores (Figure 3). We believe that the optimal intermediate excitation wavelength is blue shifted slightly compared to the 293T imaging experiments because higher energy was required to resolve the finer microtubule structures. Simultaneous CLSM and ME-MPM images exhibited comparable signal resolution in the green and red emission channels (Figure 3a-b, green and red arrows, respectively) and both showed slight bleed-through of green fluorescence signal in the red emission channel (Figure 3a-b, dimmer green arrows). Cross-excitation of Alexa Fluor 594 by the green excitation source in ME-MPM was predicted based on its TPA spectrum (Figure 1c), but was dim compared to the true red and green signal intensities (Additional file 3).

Using SE-MPM at 950 nm, green signal was much more intense than red signal, thereby masking the signal in the red channel (Figure 3c). In the SE-MPM images, there was notable bleed-through of green signal into the red emission channel that was as intense as the true red signal in the sample. Unequal signal intensity and bleed-through have been reported previously for SE-MPM [11]. Therefore, ME-MPM enhanced the spectral separation of green and red fluorescing probes in finer subcellular structures over SE-MPM and was comparable to simultaneous CLSM.

A further limitation of SE-MPM that came up frequently in our measurements was the lengthy processes involved in optimizing the intermediate excitation wavelength for a given set of fluorophores in a sample. For each pair of fluorophores, finding the optimal intermediate excitation wavelength is a laborious process and often requires the use of finely tuned VBFs to optimize fluorescence emission separation as well as access to the entire set of fluorescent small molecules or proteins in order to design a pair that can be used for a given sample [12,13]. We found that even after an optimal wavelength had been identified for a given pair of fluorophores, this wavelength ultimately depended on the concentration and expression levels of each fluorophore in the sample, as was noted for the 293T and Cos7 cell experiments. The ME-MPM system allowed us to quickly adjust the half-wave plates in the optical path to achieve equal fluorescence signal intensity with minimal bleed-through for green and red dyes. Therefore, it was simple to optimize imaging conditions regardless of expression levels and fluorescence intensity of the two fluorophores in the sample, much like the level of control in sequential CLSM.

### Multi-color time-lapse imaging in photosensitive samples

CLSM has not been sufficient to perform long time-lapse imaging experiments in living, photosensitive tissues because it requires repeated broad exposure of the specimen to high energy UV and visible excitation light [4]. Previously, we have noted considerable phototoxicity in acute brain slices over 12 hours of fluorescence imaging using standard CLSM systems (data not shown). To test the application of our ME-MPM system for multi-color time-lapse imaging of photosensitive cells, acute tissue slices were prepared from an embryonic brain in which cells had been dual-labeled with hrGFP and DsRedII via *in utero *electroporation [17].

**Figure 4:**
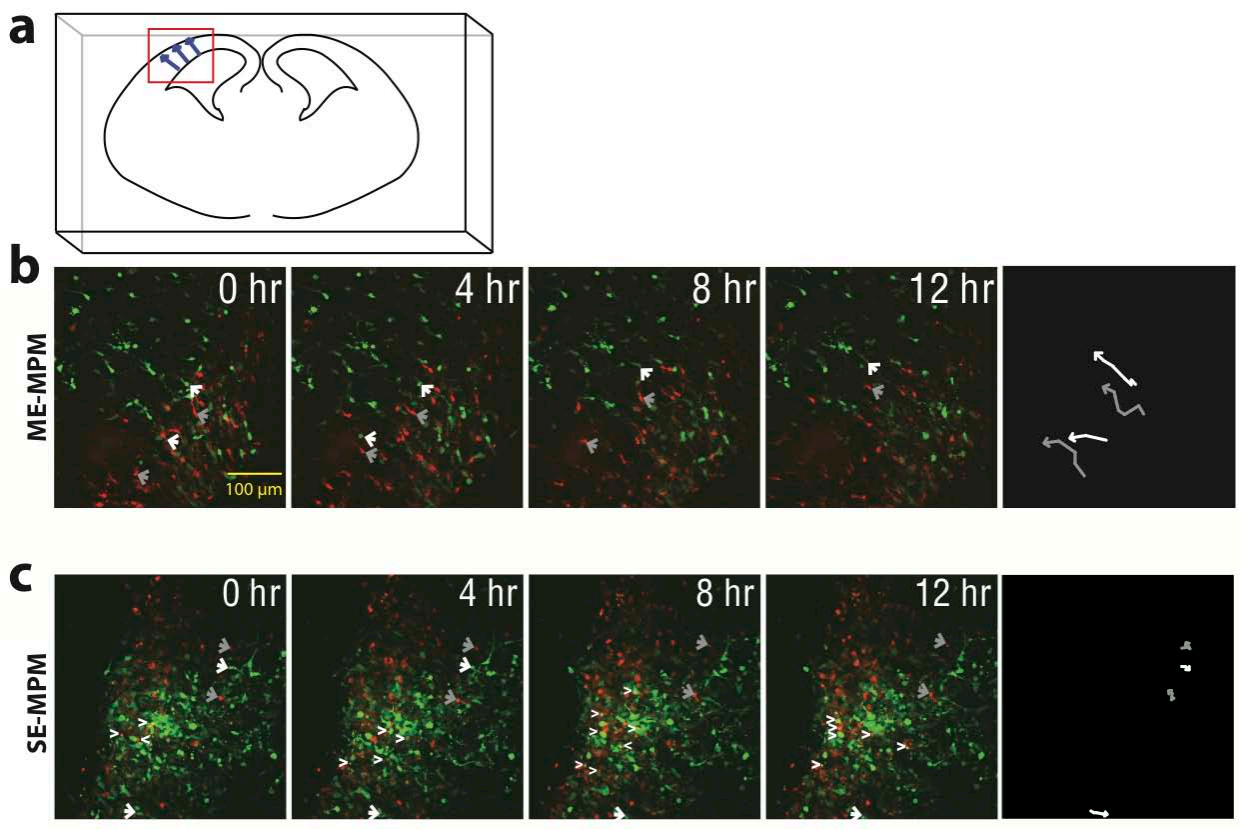
**Me-MPM improved signal-to-noise during time-lapse imaging of photosensitive tissue samples**. Images of 250-μm acute brain slices prepared on embryonic day 17 from a mouse embryo that underwent consecutive two-color *in utero *electroporation of hrGFP and DsRedII to generate two distinct groups of red or green fluorescing cells in the cortex, schematically represented in **a**. Therefore, any yellow signal should correspond to signal bleed-through (verified empirically using CLSM on independent samples). Tissue slices were imaged using simultaneous ME-MPM at 920 nm and 1015 nm (**b**) or SE-MPM at 970 nm (**c**). Note that bleed-through of green signal in the red emission channel was higher for SE-MPM (white arrowheads). Two green and two red cells for each experiment were traced at 1-hour intervals over the 12-hour imaging window (white and grey arrows, respectively) and their movement is shown (right panel in **b **and **c**). Note that cell movement was robust for green and red neurons that were imaged using ME-MPM. In similar samples, CLSM has previously been shown to cause more photo-induced cell death [2].

We adapted the standard single color electroporation by separating two single color electroporations by one day, thereby generating two distinct groups of fluorescing cells expressing only one of the two fluorescent proteins within the cortex. A key feature of this application is that all such electroporated cells can be traced as they migrate toward the cortex in subsequent days by fluorescence MPM [18]. Migration of green and red populations of neurons within the mouse cortex of E17 brain slices was imaged by collecting 3D z-stacks every 10 minutes for 12 hours using ME-MPM at 920 nm and 1015 nm on the FV300 upright microscope (schematic in Figure 4a). We chose to excite DsRedII at 1015 nm instead of 1040 nm because the IR laser power was more stable at this shorter wavelength, which was crucial for longer time-lapse experiments.

Time-lapse images from an acute brain section imaged using ME-MPM revealed no apparent cross-excitation or bleed-through in the emission channels (Figure 4b, signal would appear yellow), and all cells could easily be identified as expressing one of the two fluorescent proteins (Figure 4b, Additional file 4). Representative green- and red-fluorescing cells in the imaging plane were traced over a 12-hour imaging window (white and grey arrows, respectively), and movement was represented using vector diagrams (Figure 4b right panel). Both green and red fluorescing neurons migrated radially in the field of view for all 12 hours of image collection, indicating minimal phototoxicity in the sample. Additionally, after 12 hours, there was no observable decrease in fluorescence signal intensity or photo-induced cell death. Therefore, ME-MPM provided increased viability in photosensitive cells compared to CLSM without sacrificing signal-to-noise levels or spectral separation. Whether this would hold true in other photosensitive systems has yet to be determined.

For comparison, an adjacent acute brain section of the cortex was imaged using a single, intermediate excitation wavelength at 970 nm, which we empirically found to be the optimal intermediate excitation wavelength (Figure 4c, Additional file 5). The excitation power and exposure times were adjusted to achieve comparable red signal intensity as ME-MPM (Figure 4b, Additional file 4) so that image quality and tissue viability could be directly compared. SE-MPM images had observable cross-excitation and false positive bleed-through of green fluorescence signal in the red channel (yellow cells Figure 4c). In addition, green fluorescence signal was more intense than red fluorescence signal, which slightly masked the red signal in the image series. Similar to ME-MPM, there was no observable decrease in fluorescence signal intensity over the 12 hours of data collection.

SE-MPM is the gold standard for imaging multiple fluorophores with two-photon excitation. We noted that neurons moved at least the same if not greater distances under ME-MPM, suggesting that this new method allows for increased signal-to-noise ratios and decreased false positive emission bleed-through without sacrificing cell viability. We speculate that neurons exhibited enhanced movement when imaged under ME-MPM due to faster experimental set up time and reduced exposure to excitation light before time-lapse imaging began. In the ME-MPM setup, we could simply tune each excitation source independently to optimally excite each fluorophore, similar to what is done in CLSM. However, as we noted earlier, the SE-MPM setup required systematic evaluation of excitation at several intermediate wavelengths to find the optimal wavelength to excite both fluorophores relatively equally without evoking false positive bleed-through signal, and this led to slower experimental set up times and increased exposure to excitation light before the time-lapse imaging could begin. In addition, the optimal intermediate wavelength was not easily predictable because it varied between experiments based on the relative concentration levels and expression patterns for each fluorophore. Further experiments should be performed to elucidate whether ME-MPM imaging leads to reduced photo-toxicity in standard paradigms using time-lapse imaging in live cells.

There are a number of modifications that could improve the current ME-MPM design to further enhance spectral separation and signal-to-noise levels. Presently only 50% of the combined available power from the two excitation sources could be directed to the sample due to the design of the beam combiner, which orients the beams to be anti-parallel upon exiting. This limitation was most notable when attempting to visualize fine subcellular structures at high magnification (Figure 3). The available power at the sample could be increased by replacing the beam combiner with an excitation dichroic mirror, capable of passing light below 980 nm and reflecting light above 980 nm, at the intersection of the two beams in the routing path. This modification in the light path would allow for a greater power range at the sample from the excitation sources.

The system could also be improved by using excitation lasers with a broader wavelength range. In this report the wavelength used to excite DsRedII and Alexa Fluor 594 was between 1015 nm and 1040 nm. However, some red-emitting fluorophores have peak excitation above 1040 nm ([8,9] and Figure 1b-c). Longer excitation wavelengths would increase the signal-to-noise levels in the red emission channel and could decrease the amount of light absorbed by green dyes, thereby further enhancing spectral separation. Additionally, we predict that spectral separation and signal-to-noise could be further improved in the time-lapse imaging experiment using an excitation source that is more stable at 1040 nm for long periods of data collection. Alternatively, shorter excitation pulses could also be used to improve excitation efficiency, decreasing the need for longer wavelength excitation.

We believe that ME-MPM could also be adapted for two-photon FRET experiments that require very high temporal resolution and signal separation. Such a system would be ideally designed with an electronic shuttering mechanism for sequential wavelength excitation rather than simultaneous excitation in order to effectively quantify donor and acceptor excitation within a given FRET pair. In addition, an ME-MPM configuration could be constructed with a delay stage to synchronize excitation pulses within the sample to measure fluorescence lifetime in the sample.

A potential drawback of this system is that it remains susceptible to chromatic aberration, which could make it difficult to maintain identical focal points of the two colors. However there was no observable difference in the foal plane of the green and red signal in our system (i.e., when we focused on the green signal, the red signal was also in focus and vice versa). If this problem does present itself in other imaging systems, microscope objectives are now available to correct for chromatic aberration up to 1100 nm, much like this problem was solved in multi-laser CLSM. A second potential drawback of this system compared with SE-MPM is the cost of the second IR excitation source. However, prices for IR excitation sources are continuingly dropping as the popularity of MPM increases, [4,19] further lowering the cost of the setup. In order to increase functionality in our design, 100% of either excitation source can be directed at either microscope independently, providing two independent SE-MPM setups when ME-MPM is not needed. An optimized price point could provide researchers with a simple and efficient method to study in vivo molecular dynamics without compromising tissue viability or image quality.

## Conclusions

It has been a challenge to resolve two distinct fluorescence signals simultaneously using SE-MPM, thereby making MPM a less popular choice for multi-color imaging despite the many well-known advantages of MPM over CLSM. Here, we have investigated the feasibility of ME-MPM, a simple adaptation of the standard SE-MPM system to allow for two separate sources of excitation that are optimized based on the TPA spectra for pairs of fluorescent molecules. The advantages of ME-MPM over SE-MPM were threefold. First, this technique allowed for the selection of optimal excitation wavelengths for each fluorophore that minimally excited the other, thereby enhancing signal-to-noise ratios compared to images collected using SE-MPM (Figure 2). Second, the implementation of half-wave plates and fixed Nicol prisms allowed us to easily adjust the intensity of excitation illumination from each IR laser source separately to achieve standard illumination of both fluorophores by bringing only the brightest point in the imaging field to saturation in each channel. This level of control, similar to that of CLSM, enhanced the spectral separation of the two fluorophores compared to SE-MPM and did not require the use of VBFs (Figure 3). By comparison, in SE-MPM, these adjustments to normalize signal intensity for optimal imaging conditions are simply not possible without further narrowing the VBFs, which requires compensatory increased exposure to excitation light. Finally, live cells imaged under ME-MPM remained viable after a 12-hour time-lapse experiment, suggesting that this new method allows for increased signal-to-noise ratios and decreased false positive emission bleed-through without sacrificing cell viability (Figure 4).

In the future, with optimized spectral separation, the ME-MPM system could be used to more rapidly design pairs of fluorescence probes for multi-color two-photon imaging, such as CFP/YFP or GFP/DsRed for CLSM. Similar to the tremendous gain in popularity of CLSM after the introduction of multi-color imaging, we anticipate that ME-MPM will further increase the popularity of MPM.

## Methods

### Measurement of Two-Photon Absorption Spectra and Cross Sections

For measurements of two-photon absorption (TPA) spectra, we used a linearly polarized light of a tunable femtosecond optical parametric amplifier (TOPAS, Quantronix), pumped with a 1-kHz repetition rate Ti:sapphire femtosecond regenerative amplifier (Coherent, Legend). We recorded the fluorescence of the sample solution (C ~ 10^-5 ^M in PBS pH 7.4) in a 1-cm cuvette at 90°to excitation beam as a function of the excitation wavelength with an imaging spectrometer (Triax 550, Jobin-Ivon). Fresh solutions were used in all measurements.

TPA spectra were corrected for photon flux, pulse duration, and beam spatial profile variations as a function of the excitation wavelength. Only the spectral region, where the power dependence of fluorescence signal is quadratic, was scanned and presented here. The TPA cross section (σ_2_) of the sample at one selected wavelength (740 nm) was then measured and the whole TPA spectrum was scaled to that value.

For σ_2 _evaluation, we employed a method that compares the intensities of two-photon excited fluorescence of an unknown sample and reference standard at a selected wavelength [20]. This approach only required knowing the concentrations (or extinction coefficients) of the two samples and their relative differential quantum efficiencies of fluorescence at that selected wavelength, which can be obtained in a one-photon excitation experiment. As a reference standard we used fluorescein in PBS (pH 11), for which σ_2 _= 30 GM at 740 nm [20,21].

### Optical Setup

ME-MPM was employed on an Olympus BX61-W1 upright confocal scan head equipped with FluoView 300 software (FV300) and an Olympus IX81 inverted, spectral deconvolution confocal scan head equipped with FluoView 1000 software (FV1000) using Mai Tai broad-band (BB, 710 nm - 990 nm) and Mai Tai high power (HP, 690 nm - 1040 nm) laser sources (Spectra-Physics Inc.). Half-wave plates were placed in the optical path to adjust the polarization of the excitation beam, which allowed us to control the intensity of each excitation source directed out of the subsequent polarized beam splitter (Additional file 1). Beam combiners were used to combine the two excitation beams, which oriented the beams anti-parallel to one another, before entering the microscope scan head. A list of all specialized optical components that were used in the ME-MPM system is provided in Additional file 6.

The power of the excitation light at the sample for each two-photon excitation source was measured with a power meter that was placed approximately 0.5 cm from the objective on the FV1000 inverted confocal microscope, over the entire wavelength range of the Mai Tai BB and Mai Tai HP laser sources. Measurements were taken every 20 nm at 100% power from each laser source (Additional file 2). The HP laser offered increased power over the entire wavelength range and experienced greater attenuation of power at longer wavelengths compared to the BB laser.

### Fixed Cell Preparation and Imaging

293T cells (ATTC, Rockville, MD) were cultured in OPTI-MEM medium (Gibco) with 10% FBS (vol:vol) and penicillin/streptomycin. Three days after subculturing, 293T were transfected with hrGFP-C2 (Stratagene) or DsRedII-C1 (Clontech) DNA. One day after transfection, both groups of cells were washed in PBS to remove the transfectant and then mixed in culture at a 1:1 ratio. Twelve hours after mixing, cells were fixed in 4% PFA (vol:vol in PBS) for 10 minutes and then cover-slipped for fluorescence imaging. 293T cells were imaged using a 60X/1.42 n.a. oil objective on the FV1000 inverted microscope with VBFs set to 486 - 516 nm and 606 - 706 nm for green and red emission channels, respectively. These settings were selected to minimize false-positive bleed-through between the two emission channels while preserving adequate signal in both the green and red channels. All measurements were done at the same scan settings. 293T cells were also imaged on the FV1000 inverted microscope by sequential CLSM using the 488-nm and 543-nm laser lines under identical VBF settings.

Cos7 cells (ATTC, Rockville, MD) were cultured in OPTI-MEM medium (Gibco) with 10% FBS (vol:vol) and penicillin/streptomycin. Three days after subculturing, cells were fixed in 4% PFA (vol:vol in PBS) for 10 minutes. The cells were then blocked in bovine serum albumin (BSA 0.45% wt:vol), Triton X-100 (0.4% vol:vol), and normal donkey serum (1% vol:vol) in PBS for one hour, stained with a monoclonal anti-mouse tubulin-FITC conjugated antibody (1:50, Sigma) and an Alexa Fluor 594-phalloidin conjugated antibody (1:40, Invitrogen) for one hour, and cover-slipped for fluorescence imaging. Cos7 cells were imaged using a 60X/1.40 n.a. oil objective on the FV300 upright microscope. Cos7 cells were also imaged on the FV1000 inverted microscope by simultaneous CLSM using the 488-nm and 543-nm laser lines.

Cells that were selected for imaging were chosen at random in locations where the juxtaposition of hrGFP and DsRedII expressing cells were in the same field of view in the case of the 293T cells, or for large, flat, well-defined cells in the case of the Cos7 cells.

### Slice Preparation and Imaging

Plasmids hr-GFP-C2 (5 μg/μL, Stratagene) and pDsRedII-C1 (7. 5 μg/μL, Clontech) were injected and then electroporated into the cortex of embryonic mice (ICR strain) at E14 and E15, respectively, using the ECM-830 Electro Square Porator (BTX Division of Genetronics). Transverse cortical slices (250 μm thick) were prepared at E17 in 3% agarose (ultrapure, low melting temperature) dissolved in complete HBSS (1× HBSS, 2.5 mM HEPES pH 7.4, 30 mM D-glucose, 1 mM CaCl_2_, 1 mM MgSO_4_, 4 mM NaHCO_3_) using the Vibratome 1000 Plus Sectioning System (Ted Pella, Inc.). Slices from each mouse were transferred onto a 60-mm plate, immersed in media containing collagen, and incubated at 37°C with 5% CO_2 _for 30 to 45 minutes to allow the collagen to solidify. N2 culture media (DMEM, penicillin/streptavidin, N2, HEPES, L-glutamate) was added to the plate to cover the entire collagen surface and the plate was incubated at 37°C with 5% CO_2 _for 30 to 45 minutes before imaging. Slices were imaged on the FV300 upright microscope with a LUMPLFL 20×/0.96 n.a. water objective. For ME-MPM measurements, excitation at 920 nm and 1015 nm was used and the half-wave plates in the optical path for both excitation sources were adjusted to keep the signal intensity equal for green and red cells. For SE-MPM measurements, excitation at 970 nm was used. All experiments were performed at 37°C in N2 culture media covered with a thin layer of mineral oil to prevent media evaporation. Brain sections showing adequate electroporation of both populations of cells were chosen for imaging. Images were acquired as z-stacks every 10 minutes for 12 hours.

## Competing interests statements

The authors declare competing financial interests. The authors are co-inventors on a patent application related to the method described in this publication.

## Authors' contributions

MB contributed to the design of the optical system, prepared the samples for imaging, performed experiments, and composed the figures. MD and NM measured two-photon absorption spectra. AR developed the experimental method for two-photon absorption measurements. BB contributed to the design of the optical system. JG and BB conceived the method and edited the manuscript. All authors read and approved the final manuscript.

## Supplementary Material

Additional file 1 **Rational for the use of three sets of half-wave plates and two fixed prisms**. This note provides a detailed description of our rational for using three sets of half-wave plates and two fixed prisms in the optical path for ME-MPM. Click here for file

Additional file 2 **Power curves for the Mai Tai broad-band (BB) and Mai Tai high power (HP) laser sources**. Power curves were measured at the focal plane using the 60×/1.42 n.a. oil objective on the FV1000 inverted confocal microscope. Points and bars represent the average and standard deviation from five measurements, respectively. Click here for file

Additional file 3 **Red and green emission signal generated from each ME-MPM excitation source**. Images were collected after diverting either the green (labeled 1040 nm) or the red (labeled 920 nm) excitation laser before it entered the beam combiner and were collected using identical imaging settings as described for Figure 2 (293T cells, **a-b**) and Figure 3 (Cos7 cells, **c-d**). Click here for file

Additional file 4 **Time-lapse video of an acute brain slice that was imaged using ME-MPM**. Time-lapse imaging of a 250-μm acute slice prepared on embryonic day 17 from a mouse embryo that underwent consecutive two-color *in utero *electroporation of hrGFP and DsRedII (described in the text) using ME-MPM at 920 nm and 1015 nm. Images were collected every 10 minutes for 12 hours. Note that cell movement was robust for green and red neurons imaged using ME-MPM. Click here for file

Additional file 5 **Time-lapse video of an acute brain slice that was imaged using SE-MPM**. Time-lapse imaging of a 250-μm acute slice prepared on embryonic day 17 from a mouse embryo that underwent consecutive two-color *in utero *electroporation of hrGFP and DsRedII (described in the text) using SE-MPM at 970 nm. Images were collected every 10 minutes for 12 hours. Note that cross-excitation and bleed-through were problematic (yellow cells), and cell movement was compromised in this experiment. Click here for file

Additional file 6 **A list of specialized optical components that were used in this ME-MPM system**. We have provided a list of all specialized optical components that were used in this ME-MPM system for investigators that would like to add the ME-MPM capability to their imaging system. All other optical components were standard. A typical system should have high sensitivity fluorescence detection capability from 400 nm to 750 nm, and IR optical components should have good transmission in the IR range from 690 nm to 1100 nm. Click here for file
