# Supplementary material for: Simultaneous multiple-excitation multiphoton microscopy yields increased imaging sensitivity and specificity
Source: BMC Biotechnol. 2011 Mar 2;11:20. doi: 10.1186/1472-6750-11-20 (PMC3062589; doi:10.1186/1472-6750-11-20)
Supplement: Additional file 1 — Rational for the use of three sets of half-wave plates and two fixed prisms. This note provides a detailed description of our rational for using three sets of half-wave plates and two fixed prisms in the optical path for ME-MPM. [file 1472-6750-11-20-S1.DOC]

Additional note. Rational for the use of three sets of half-wave plates and two fixed prisms.

The optical setup contains three sets of half-wave plates and Nicol prisms. The first set of half-wave plates (i) and prisms is only necessary for a setup that contains two microscopes, in this case an inverted and an upright microscope. This first set was used to send all (100%) or none (0%) of each excitation laser to either system. Therefore, in our design, this first set allows both two-photon excitation sources to be used independently for SE-MPM or jointly for ME-MPM on either microscope, which greatly broadens the versatility of this technique. The second set of half-wave plates (ii) and Nicol prisms was used to modulate the amount of laser light that is sent into the beam combiner from each two-photon excitation sources independently, which will depend on the relative expression or staining pattern of the red and green dyes. To achieve this for a given sample, we blocked the red excitation laser and adjusted the half-wave plate (ii) in the green optical path to just reach saturation of the green signal, and we repeated this for the half-wave plate (ii) in the red optical path when the green excitation laser was blocked. Because the two excitation lasers exit the beam combiner orthogonal to each other, the ratio of the green and red signal could be modulated with respect to the other using the third set of half-wave plates (iii) and Nicol prisms to achieve similar signal strength and signal-to-noise levels in both emission channels. This third set is not necessary for the ME-MPM setup, but we found it to be very useful in practice to optimize the signal in both channels.
