# Supplementary figures and images for: Simultaneous multiple-excitation multiphoton microscopy yields increased imaging sensitivity and specificity
Source: BMC Biotechnol. 2011 Mar 2;11:20. doi: 10.1186/1472-6750-11-20 (PMC3062589; doi:10.1186/1472-6750-11-20)

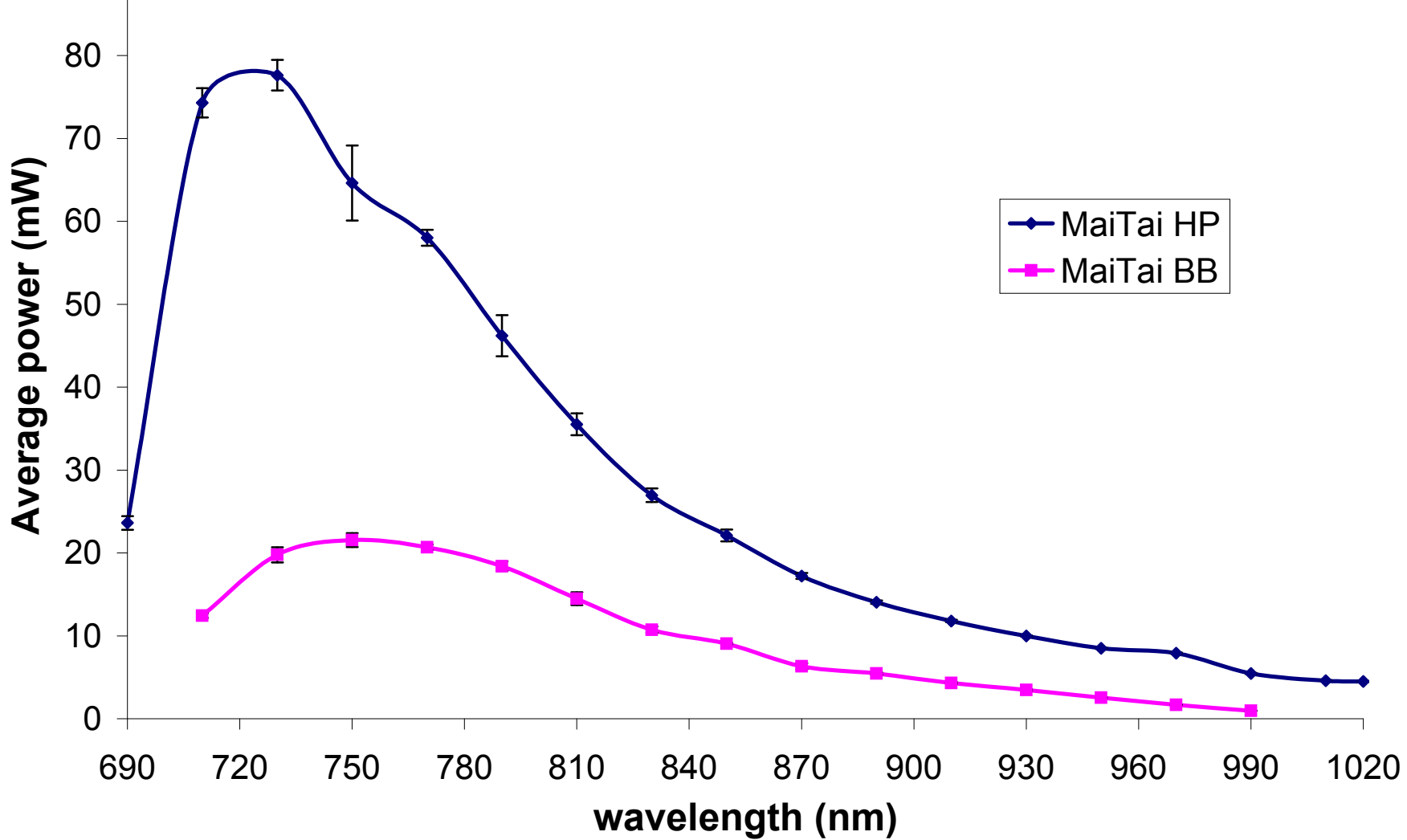

Supplement: Additional file 2 — Power curves for the Mai Tai broad-band (BB) and Mai Tai high power (HP) laser sources. Power curves were measured at the focal plane using the 60×/1.42 n.a. oil objective on the FV1000 inverted confocal microscope. Points and bars represent the average and standard deviation from five measurements, respectively. [file 1472-6750-11-20-S2.PDF]

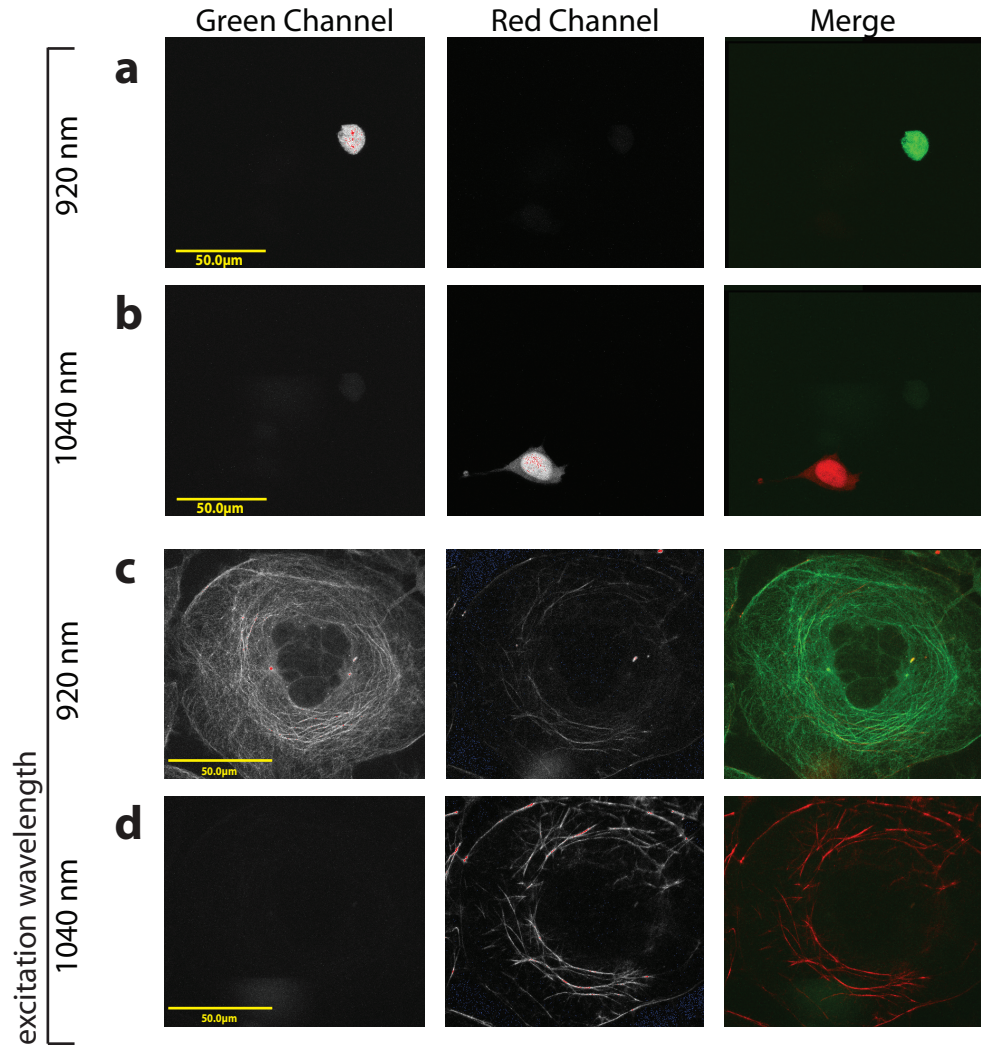

Supplement: Additional file 3 — Red and green emission signal generated from each ME-MPM excitation source. Images were collected after diverting either the green (labeled 1040 nm) or the red (labeled 920 nm) excitation laser before it entered the beam combiner and were collected using identical imaging settings as described for Figure 2 (293T cells, a-b) and Figure 3 (Cos7 cells, c-d). [file 1472-6750-11-20-S3.PDF]
