# Supplementary material for: Simultaneous multiple-excitation multiphoton microscopy yields increased imaging sensitivity and specificity
Source: BMC Biotechnol. 2011 Mar 2;11:20. doi: 10.1186/1472-6750-11-20 (PMC3062589; doi:10.1186/1472-6750-11-20)
Supplement: Additional file 6 — A list of specialized optical components that were used in this ME-MPM system. We have provided a list of all specialized optical components that were used in this ME-MPM system for investigators that would like to add the ME-MPM capability to their imaging system. All other optical components were standard. A typical system should have high sensitivity fluorescence detection capability from 400 nm to 750 nm, and IR optical components should have good transmission in the IR range from 690 nm to 1100 nm. [file 1472-6750-11-20-S6.PDF]

Additional Table 1: Specialized optical equipment needed to build the ME-MPM system

**Optical component**	**Model Number**
Nicol prism (polarizing beamsplitter)	Newport 05FC16PB.5
Half-wave plates	Newport 10RP52-2
Single axis, hand-held, stepper motor controller/driver (to modulate the rotation of each half- wave plate)	Newport NSC200
Universal rotator (to rotate each half-wave plate)	Newport NSR1
Beam combiner	Newport 10GL08AR.16
Power meter	Coherent 1098293
